# Prediction of EEG Seizures in Critically Ill Children

**DOI:** 10.15844/pedneurbriefs-34-23

**Published:** 2020-12-18

**Authors:** Hesham T. Ghonim, Arayamparambil C. Anilkumar

**Affiliations:** 1 Upstate Medical University, Syracuse, NY

**Keywords:** Seizures, Encephalopathy, Critical Care, EEG monitoring, Pediatric, Neurology

## Abstract

In a prospective observational study, investigators from the Children’s Hospital of Philadelphia devised a predictive model for capturing electrographic seizures in critically ill pediatric patients.

In a prospective observational study, investigators from the Children’s Hospital of Philadelphia devised a predictive model for capturing electrographic seizures in critically ill pediatric patients. The study included a total of 719 children admitted to the intensive care unit of a quaternary care institution. Neonates below 30 days and patients who presented with status epilepticus were excluded. Continuous electroencephalographic (EEG) monitoring (CEEG) was performed for abnormal movements, encephalopathy, or seizures. Electrographic seizures were captured in 26% (184 children) of the cohort, and 6% (44 children) diagnosed with electrographic status epilepticus. Variables included age, etiological category of acute encephalopathy (structural, non-structural, or epilepsy-related), clinical seizures before initiation of CEEG, EEG background, and epileptiform abnormalities. The following factors were associated with a statistically significant difference in the odds of capturing electrographic seizures; Patients < 1 year of age, seizures before CEEG, epileptiform discharges during the initial 30 minutes of the recording, and EEG showing a slow disorganized, discontinuous, or burst suppression background. The optimal, most inclusive model had a sensitivity of 92% with a negative predictive value of 93%. [1]

COMMENTARY. Multiple previously published studies indicated the high risk of electrographic seizures in encephalopathic children admitted to the critical care units [2,3]. Seizures, if untreated, could be detrimental to the neurological outcome of those patients. Unfortunately, the availability of CEEG is variable from one institution to another based on resources, including staffing and available equipment. The authors took a welcomed initiative to provide a risk assessment tool to determine critically ill patients at risk for electrographic seizures, which would benefit from CEEG.

Based on the reviewed study results, if the devised predictive model was utilized to be most sensitive, Fung et al. concluded that CEEG would be done in all patients with epileptiform discharges in the first 30 minutes (for example, on a routine EEG). Stratifying patients further can be done based on their age, being younger or older than one year. To elaborate, CEEG would be done in those younger than one year except if all the factors as mentioned earlier associated with higher statistically significant odds ratios were absent; no clinical seizures, no epileptiform discharges during the initial 30 minutes of the recording, and normal or attenuated EEG background. On the other hand, CEEG would not be done in patients older than one year with non-structural etiologies to their encephalopathy and normal EEG background regardless of a clinical seizure before monitoring. Additionally, CEEG would not be done in patients older than one year who carry a diagnosis of epilepsy with no clinical seizures on presentation and normal or attenuated EEG background. Finally, CEEG would not be done in patients older than one year with clinical seizure and normal/attenuated EEG background or without a clinical seizure and slow disorganized EEG background.

Adult seizure prediction models, for example, 2HELPS2B score by Struck et al., gave more weight to electrographic elements on the initial EEG with a focus on hospitalized rather than critically ill patients [4)]. Children with critical illness and encephalopathy have different EEG patterns as opposed to their adult counterparts. With the addition of clinical acumen and variables not accounted for in the study (e.g., type of epilepsy and intractability), the ability to prioritize critically ill children to CEEG rather than a briefer EEG or clinical monitoring could be a powerful addition to a neurologist’s toolbox and especially significant in institutions with more limited resources.

## Disclosures

The authors have declared that no competing interests exist.

## References

[1] Fung FW, Jacobwitz M, Parikh DS, Vala L, Donnelly M, Fan J (2020). Development of a model to predict electroencephalographic seizures in critically ill children. Epilepsia.

[2] Abend NS, Wusthoff CJ, Goldberg EM, Dlugos DJ (2013). Electrographic seizures and status epilepticus in critically ill children and neonates with encephalopathy. Lancet Neurol.

[3] Yang A, Arndt DH, Berg RA, Carpenter JL, Chapman KE, Dlugos DJ (2015). Development and validation of a seizure prediction model in critically ill children. Seizure.

[4] Struck AF, Tabaeizadeh M, Schmitt SE, Ruiz AR, Swisher CB, Subramaniam T (2020). Assessment of the validity of the 2HELPS2B score for inpatient seizure risk prediction. JAMA Neurol.

